# Bayesian inference of the number of factors in gene-expression analysis: application to human virus challenge studies

**DOI:** 10.1186/1471-2105-11-552

**Published:** 2010-11-09

**Authors:** Bo Chen, Minhua Chen, John Paisley, Aimee Zaas, Christopher Woods, Geoffrey S Ginsburg, Alfred Hero, Joseph Lucas, David Dunson, Lawrence Carin

**Affiliations:** 1 Electrical and Computer Engineering Department, Duke University, Durham, NC, USA; 2 Institute for Genome Sciences & Policy, Department of Medicine Duke University, Durham, NC, USA; 3 Electrical & Computer Engineering Department, University of Michigan, Ann Arbor, MI, USA; 4 Statistics Department, Duke University, Durham, NC, USA

## Abstract

**Background:**

Nonparametric Bayesian techniques have been developed recently to extend the sophistication of factor models, allowing one to infer the number of appropriate factors from the observed data. We consider such techniques for sparse factor analysis, with application to gene-expression data from three virus challenge studies. Particular attention is placed on employing the Beta Process (BP), the Indian Buffet Process (IBP), and related sparseness-promoting techniques to infer a proper number of factors. The posterior density function on the model parameters is computed using Gibbs sampling and variational Bayesian (VB) analysis.

**Results:**

Time-evolving gene-expression data are considered for respiratory syncytial virus (RSV), Rhino virus, and influenza, using blood samples from healthy human subjects. These data were acquired in three challenge studies, each executed after receiving institutional review board (IRB) approval from Duke University. Comparisons are made between several alternative means of per-forming nonparametric factor analysis on these data, with comparisons as well to sparse-PCA and Penalized Matrix Decomposition (PMD), closely related non-Bayesian approaches.

**Conclusions:**

Applying the Beta Process to the factor scores, or to the singular values of a pseudo-SVD construction, the proposed algorithms infer the number of factors in gene-expression data. For real data the "true" number of factors is unknown; in our simulations we consider a range of noise variances, and the proposed Bayesian models inferred the number of factors accurately relative to other methods in the literature, such as sparse-PCA and PMD. We have also identified a "pan-viral" factor of importance for each of the three viruses considered in this study. We have identified a set of genes associated with this pan-viral factor, of interest for early detection of such viruses based upon the host response, as quantified via gene-expression data.

## I. Background

When performing gene-expression analysis for inference of relationships between genes and conditions/phenotypes, one typically must analyze a small number of samples, each composed of expression values from tens of thousands of genes. In this setting the observed data is **X **∈ ℝ^*p*×*n*^, where each column corresponds to one of *n *samples, quantifying the associated gene-expression values for all *p *genes under investigation. We typically must address the "large *p*, small *n*" problem [1], in which often *n *≪ *p*. Therefore, to yield reliable inference, one must impose strong restrictions on the form of the model.

When developing regression and classification models for gene-expression data, a widely employed assumption (restriction) is that the model parameters are sparse, implying that only a small subset of the genes are important for prediction. If only a small set of genes (≪ *p*) are responsible for differences in disease groups, then reliable inference may often be performed even when *n *≪ *p*. Example approaches that have taken this viewpoint are lasso [2], the elastic net [3], and related Bayesian approaches [4]. In fact, sparse regression and classification algorithms are widely used in many statistics and machine-learning applications, beyond gene analysis [5-7].

An important research direction for gene-expression analysis, and many other applications, involves the use of factor models [8-11]. To address the "large *p*, small *n*" problem, sparseness is again imposed, now typically on the factor loadings. Specifically, in an unsupervised setting the data are assumed to satisfy

(1) X=AS+E

where **A **∈ ℝ^*p*×*r*^, **S **∈ ℝ^r×n ^and **E **∈ ℝ^p × n^; if covariates are available they may also be considered in the model [11], with none assumed here. Note that here and henceforth we assume that the gene-expression data are centered in advance of the analysis; otherwise, there should be an intercept added to the model. Considering the *j*th sample, ***x**_j _*, corresponding to the *j*th column of **X**, the model states that ***x**_j _*= A***s**_j _*+ ***e**_j _*, where ***s**_j _*and ***e**_j _*are the *j*th columns of **S** and **E**, respectively.

The columns of **A **represent the factor "loadings", and rows of **S **are often called factors. To address the fact that *n *≪ *p*, researchers have typically imposed a sparseness constraint on the columns of **A **[11], with the idea that each column of **A **should ideally (in the gene application) correspond to a biological "pathway", which should be defined by a relatively small number of correlated genes. Within Bayesian formalisms, the sparse columns of **A **are typically imposed via spike-slab-like priors [1], [11], or alternatively via shrinkage (*e.g*., Student-t [6]) priors. Several non-Bayesian approaches have also been introduced, including sparse-PCA [12] and the related Penalized Matrix Decomposition (PMD) [13].

A problem that is receiving increased attention in factor-analysis-based approaches is a means of defining an appropriate number of factors (*i.e*., to infer *r*). The non-Bayesian approaches are often sequential, and one may infer *r *by monitoring the error ||*E||_F _*as a function of iteration number [12], [13]. In many previous Bayesian approaches *r *has just been set [11], and presumably many non-biologically-relevant factor loadings are inferred. A computationally expensive reverse-jump MCMC approach has been developed [14], with computational efficiency improved in [15] while also considering a default robust prior specification. Perhaps the most widely employed approach [16-18] for choosing *r *is the Bayesian information criteria (BIC). A disadvantage is that conditioning on a fixed choice of the number of factors ignores uncertainty and the BIC is not well justified in hierarchical models, as the number of parameters is unclear.

There has been recent interest in applying nonparametric Bayesian methods [8], [9] to infer *r *(in fact, a posterior distribution on *r*), based on the observed data **X**. An example of recent research in this direction employs the Indian Buffet Process (IBP) [19], [20]. In this paper we also consider the Beta Process (BP), recognizing that the BP and IBP are closely linked [21], [22].

For data sets with very large *p (e.g*., 10,000 or more), computational efficiency is of major practical importance. In previous use of nonparametric Bayesian methods to this problem, a Gibbs sampler has typically been employed [11]. The BP-based formulation admits a relatively simple variational Bayesian (VB) [23] approximation to the posterior, which is considerably faster than Gibbs sampling. An advantage of a VB analysis, in addition to speed, is that convergence is readily monitored (for the Gibbs sampler there are typically challenges when assessing convergence). We perform a comparison of the difference in inferred model parameters, based on VB and Gibbs analysis.

The specific data on which the models are employed correspond to gene-expression data from recent viral challenge studies. Specifically, after receiving institutional review board (IRB) approvals from Duke University, we performed three separate challenge studies, in which individuals were inoculated with respiratory syncytial virus (RSV), Rhino virus, and influenza. Informed consent was used in all studies. Using blood samples collected sequentially over time, we have access to gene-expression data at pre-inoculation, just after inoculation, and at many additional time points up to the point of full symptoms (such data were collected on all subjects, although not all became symptomatic). Using these data, we may investigate time-evolving factor scores of samples, to examine how the response to the virus evolves with time. Of particular importance is an examination of the factors of importance for individuals who became symptomatic relative to those who did not. In the factor analysis we consider data individually for each of the three viruses (at all times), as well as for all three viruses in a single analysis (seeking pan-viral factors). Results are generated based on nonparametric Bayesian approaches to factor analysis, employing the Beta Process, the Indian Buffet Process, and a related sparseness-constrained pseudo-SVD construction (a Bayesian construction of sparse-PCA [12]). We also make comparisons to the non-Bayesian Penalized Matrix Decomposition (PMD) [13].

## II. Results

### A. Brief summary of models

We first provide a brief intuitive explanation of the workings of the different Bayesian models considered. These models are built around the Indian buffet process (IBP) [19], so named for the following reason. In the factor model of (1), the columns of **A **represent factor loadings in which the gene-expression values for sample *j *are expressed: ***x**_i _*= A***s**_j _*+ ***e**_j _*. One construction of the IBP constitutes a set of candidate columns of **A**, and these are termed "dishes" at an Indian "buffet". Each of the *n *samples {***x**_j_*}_*j *= 1,*n *_correspond to "customers" at the buffet; each customer selects a subset of dishes from the buffet (*i.e*., selects a subset of candidate columns of A). The IBP is constructed such that the more a particular dish (column of A) is used by a subset of customers {***x**_j_*}*_j _*_= 1,_*_n_*, the more probable it is that it will be used by other customers. Thus, the IBP imposes the idea that many of the samples {***x**_j_*}*_j _*_= 1,_*_n _*will utilize the same subset of columns of **A**, but each sample may also utilize idiosyncratic factor loadings, representing unique characteristics of particular samples. The IBP construction does not impose a total number of factors for the data {***x**_j_*}*_j _*_= 1,*n*_, with this number inferred by the analysis. Thus, the IBP is a natural Bayesian method for inferring the number of factors appropriate for representing all observed data {***x**_j_*}*_j _*_= 1,*n*_. A convenient means of implementing the IBP employs the Beta process (BP) [21].

There are multiple ways in which one may utilize the IBP/BP within the factor model, with three such methods considered here: (*i*) the BP is applied to the factor scores **S **(termed below the BP construction), (*ii*) the IBP is employed on the factor loadings **A **[8] (termed below the IBP construction), and (*iii*) a BP-like construction is employed to implement a Bayesian construction of a singular-value decomposition of **X **(termed below the pseudo-SVD construction). To realize the approximate posterior density function for the parameters of these models, we have considered both MCMC and VB computational methods. The specifics of the BP, IBP and pseudo-SVD methods, as well as computational details, are provided in Section IV.

### B. Synthesized Data

The first validation example we considered was taken from [8]. In this example the gene-factor connectivity matrix of an E-coli network is employed to generate a synthetic dataset having 100 samples of 50 genes and 8 underlying factors. The data had additive white Gaussian noise with a signal-to-noise-ratio of 10. For this very small-scale example we considered all three Bayesian methods (BP, IBP and pseudo-SVD); in each case we considered both MCMC and VB methods for inferring the posterior density function. We also considered the non-Bayesian PMD and sparse-PCA [13], [24]. All methods performed well in uncovering the proper number of factors, and in capturing the proper genes associated with each factor. For brevity we do not provide further details on this example. While it is worthy of consideration because it was considered in related published research [8], its small-scale nature (only 50 genes) makes it less relevant for the large-scale real application we consider below. Therefore, in the next synthetic example we consider a much larger-scale problem, and consequently for that problem we were unable to test against the IBP method.

The synthetic data were generated as follows. A total of *p *= 10, 000 features ("genes") are employed, and the expression value for these *p *genes was constituted using five factors (*r *= 5) plus a noise term **E **(*i.e*., via the model in (1)). For each of the five factors, a unique set of 50 genes were selected and were given a factor-loading value of one. In addition, ten more genes were selected, with these shared among all five factors (again with unit-amplitude contribution to the factor loadings). Thus, a total of 260 genes contributed non-zero loadings to at least one of the five factors. For all other genes the factor-loading contribution was set to zero. The above construction defines the sparse matrix **A **in (1). The components of **S **∈ ℝ^*r*×*n*^, for *n *= 150 samples, are drawn i.i.d. from N (0, 1). The elements of the noise matrix **E **are drawn i.i.d. from $N(0,\alpha_{0}^{-1})$. The data **X **were then utilized within the various factor-analysis models, with the data-generation process repeated 100 independent times (100 different X), with mean and standard-deviation results presented on the inferred model parameters (discussed below), based on the 100 runs.

We consider a range of noise variances 1/*α*_0 _to constitute **E**, to address model performance as a function of the signal-to-noise ratio (SNR). As one definition of SNR, one may consider the average energy contributed from a non-zero gene to a particular factor, relative to the energy in the noise contribution for that gene, from E. Based on the fact that the non-zero components of **A **have unit amplitude, and the components of **S **are drawn from N (0, 1), on average (across many samples) the energy contributed by a non-zero gene to a particular factor is one. The average noise energy contributed to each gene is 1/*α*_0_. Hence, the ratio of these two quantities, *α*_0_, may be considered as a measure of SNR. Other measures of SNR may be defined with respect to this model, each of which will be defined in terms of *α*_0_.

**Figure 1:**
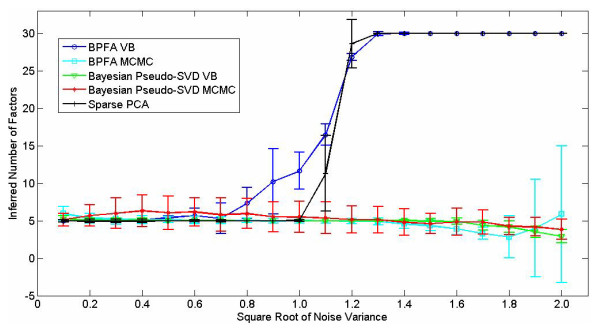
**Number of inferred factors for various algorithms, as applied to synthesized data (for which there are five factors used to generate the data)**. The data were generated randomly 100 times, with mean and standard deviation depicted. The horizontal axis denotes $1/\sqrt{\alpha_{0}}$

In Figure 1 are presented the average number of inferred factors and the associated standard deviation on this number, for the BP and pseudo-SVD models. We also compare to the sparse-PCA model in [12]. The integer *K *represents the truncation level in the models, defining the maximum number of columns of **A **considered for analysis, from which *r *≤ *K *columns are inferred as needed to represent the data X. This is discussed in detail in Section IV. In these examples the models were each truncated to *K *= 30 factors. Consequently, when 30 factors are used, the models have effectively failed, since the true number of factors is 5 and 30 is the maximum allowed within the model, given the truncation level under consideration. The MCMC results are based upon 2000 burn-in iterations and 1000 collection iterations (the results are similar when 10,000 collection iterations are employed). Results are shown as a function of the standard deviation of the noise, $1/\sqrt{\alpha_{0}}$. The sparse-PCA model works well up to the point that the noise variance equals the amplitude of the non-zero values in **A **(approximate SNR of one), while most of the Bayesian methods infer the proper number of factors to a higher level of noise.

**Figure 2:**
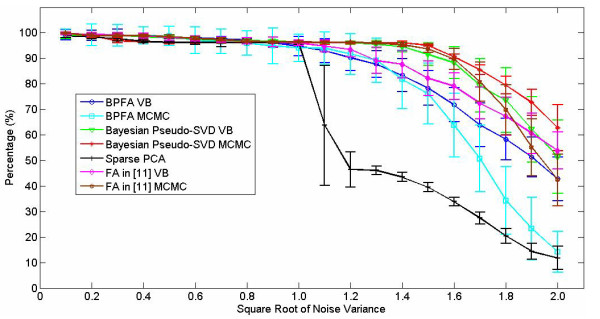
**Considering the same data as in Figure 1, for which 260 genes had non-zero contributions to the factor loadings used for data generation, we plot the percentage of the *inferred *most important 260 genes that are consistent with the true genes used for data generation**. A value of 100% implies that all of the inferred top-260 genes are consistent with those used for data generation. The data were generated randomly 100 times, with mean and standard deviation depicted.

In Figure 2 we examine how meaningful the inferred factor loadings are. Specifically, recall that the data are based upon 260 unique genes that contribute to the factor loadings. Based on the inferred factor loadings, we rank the genes based upon their strength in the loadings. We then rank the genes from 1 to 260, based on the above strength, and examine the percentage of the top 260 *inferred *genes are consistent with truth. Considering Figure 2, all of the Bayesian methods perform well in this task, up to a noise standard deviation of approximately 1.3, while sparse-PCA performs degrades quickly beyond standard deviations of one (for SNR values below one). Note that we also consider the Bayesian factor analysis model in [11]; we did not consider this method in Figure 1 because it does not have a mechanism for estimating *r*-we simply set *r *= *K *in this analysis, using the same *K *= 30 as employed for the other Bayesian methods. In [11] the authors only considered an MCMC implementation, where here we consider both MCMC and VB inference for this model; further, here we have employed a Student-t prior on the components of the factor loading matrix **A**, where in [11] a spike-slab prior was employed.

Concerning sparse-PCA [12] (and PMD, not shown), every effort was made to optimize the model parameters for this task. Our experience is that, while sparse-PCA and PMD [13] are very fast algorithms, and generally quite effective, they are not as robust to noise as the Bayesian methods (the Bayesian methods are also less sensitive to parameter settings). It is possible that the sparse-PCA and PMD results could be improved further if the model parameters are optimized separately for each noise level (and the Bayesian results may also be improved with such tuning). However, the model parameters were fixed for all noise variances considered (since the noise variance is often not known *a priori*, with the sparse-PCA carefully tuned to achieve the best results in such a circumstance.

We also performed additional simulated examples of the type discussed above, the details of which are omitted for brevity. In those experiments the different genes did not have the same noise variance. The Bayesian methods, which as indicated above infer the noise variance separately for each gene, performed as well as in Figures 1 and 2. However, the sparse-PCA and PMD models performed relatively poorly in this case, since they assume the same noise variance for all genes. The assumption of a constant noise variance for each gene may not be as appropriate for real data.

### C. Details on Data Collections for Three Viral Challenge Studies

We considered three cohorts of healthy volunteers experimentally infected with either rhinovirus, respiratory syncytial virus (RSV) or influenza A; these three challenges were performed separately, with no overlap in the subjects. All exposures were approved by the Duke University institutional review board and conducted according to the Declaration of Helsinki. The three challenges are briefly summarized here, with further details provided in [25].

#### Human Rhinovirus cohort

We recruited 20 healthy volunteers via advertisement to participate in the rhinovirus challenge study through an active screening protocol at the University of Virginia (Charlottesville, VA). On the day of inoculation, 10^6 ^TCID50 GMP rhinovirus (Johnson and John-son) was inoculated intranasally. Subjects were admitted to the quarantine facility for 48 hours following rhinovirus inoculation and remained in the facility for 48 hours following inoculation. Blood was sampled into PAXGene™blood collection tubes at pre-determined intervals post inoculation. Nasal lavage samples were obtained from each subject daily for rhinovirus titers to accurately gauge the success and timing of the rhinovirus inoculation. Following the 48th hour post inoculation, subjects were released from quarantine and returned for three consecutive mornings for sample acquisition and symptom score ascertainment.

#### Human RSV cohort

A healthy volunteer intranasal challenge with RSV A was performed in a manner similar to the rhinovirus intranasal challenge. The RSV challenge was performed at Ret-roscreen Virology, Ltd (Brentwood, UK) using 20 pre-screened volunteers who provided informed consent. On the day of inoculation, a dose of 10^4 ^TCID50 respiratory syncytial virus (RSV; serotype A) manufactured and processed under current good manufacturing practices (cGMP) by Meridian Life Sciences, Inc. (Memphis, TN USA) was inoculated intranasally per standard methods. Blood and nasal lavage collection methods were similar to the rhinovirus cohort, but continued throughout the duration of the quarantine. Due to the longer incubation period of RSV **A**, subjects were not released from quarantine until after the 165th hour and were negative by rapid RSV antigen detection (BinaxNow Rapid RSV Antigen; Inverness Medical Innovations, Inc).

#### Influenza cohort

A healthy volunteer intranasal challenge with influenza A/Wisconsin/67/2005 (H3N2) was performed at Retroscreen Virology, LTD (Brentwood, UK), using 17 pre-screened volunteers who provided informed consent. On the day of inoculation, a dose of 106 TCID50 Influenza A manufactured and processed under current good manufacturing practices (cGMP) by Bayer Life Sciences, Vienna, Austria was inoculated intranasally per standard methods at a varying dose (1:10, 1:100, 1:1000, 1:10000) with four to five subjects receiving each dose. Due to the longer incubation period of influenza as compared to rhinovirus, subjects were not released from quarantine until after the 216th hour. Blood and nasal lavage collection continued throughout the duration of the quarantine. All subjects received oral oseltamivir (Roche Pharmaceuticals) 75 mg by mouth twice daily prophylaxis at day 6 following inoculation. All patients were negative by rapid antigen detection (BinaxNow Rapid Influenza Antigen; Inverness Medical Innovations, Inc) at time of discharge.

For each viral challenge, subjects had samples taken 24 hours prior to inoculation with virus (baseline), immediately prior to inoculation (pre-challenge) and at set intervals following challenge. For the rhinovirus challenge, peripheral blood was taken at baseline, then at 4 hour intervals for the first 24 hours, then 6 hour intervals for the next 24 hours, then 8 hour intervals for the next 24 hours, and then 24 hour intervals for the remaining 3 days of the study. For the RSV and influenza challenges, peripheral blood was taken at baseline, then at 8 hour intervals for the initial 120 hours, and then 24 hours for the remaining 2 days of the study. All results presented here are based on gene-expression data from blood samples. For the RSV and Rhino virus cases not all blood samples were converted to gene expression values, as a cost-saving measure. Hence, for these two cases the gene expression data are not sampled as finely in time as are the influenza data.

In the statistical analysis, the matrix **X **in (1) has columns that correspond to the *n *samples; *n *= *n_s_n_t_*, with *n_s _*representing the number of subjects and *n_t _*the number of sample time points. We do not impose a prior on the time-dependence of the factors scores, and uncover this time dependence via the inferred posterior distribution of factor scores S.

### D. Analysis of influenza data

The gene-expression data consisted of over *p *= 12, 000 genes, and consequently we found that the IBP approach developed in [8] was computationally intractable. We found that the VB and MCMC results were generally in good agreement for this real data, and therefore the two very distinct computational tools served to cross-validate each other. The VB and MCMC computations also required similar CPU time (for the number of Gibbs iterations considered); while the VB analysis required far fewer iterations to converge, each iteration is significantly more expensive than that associated with the Gibbs sampler.

For brevity, we here focus exclusively on MCMC solutions when considering Bayesian analysis. Results are presented using the BP and pseudo-SVD methods, as well as via PMD [13] (similar results were computed using sparse-PCA [24]). We note that the design of each the experiments involves samples from the *same *subjects observed at multiple time points (with different subjects for the three viruses). Therefore, the assumption within the models that the samples at different times are statistically independent may warrant reconsidering in future studies. This subject has been considered in related work [26], although that research assumes a known factor structure and Gaussian latent factors.

**Figure 3:**
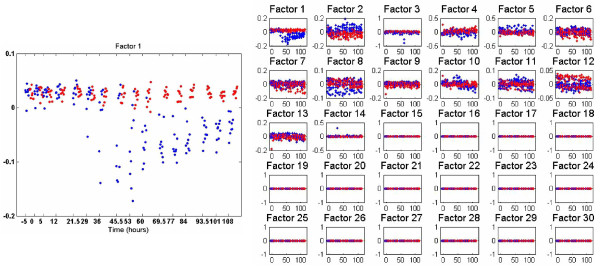
**Factor-analysis of Flu data with BP applied within design of factor scores, as discussed in Section IV-B**. The MCMC inference was based on 2000 burn-in iterations and 500 collection iterations, and factor scores are depicted for one (typical) collection sample from the Gibbs sampler. Approximately thirteen factors were inferred with non-zero factor scores (shown at right), and at left is a blow-up of the factor that most separates symptomatic (blue) from asymptomatic (red) samples. The horizontal axis denotes time in hours. The data were collected in groups, at discrete times; the results at a given time are shifted slightly along the horizontal axis with respect to one another, to enhance readability.

We first consider results based on the BP as applied to the factor scores. In these results we set *K *= 30 (recall this is the truncation level on the number of factors), and inferred approximately *r *= 13 important factors (see Figure 3); although only approximately *r *= 13 factors are used, we show the factor scores for all *K *= 30 possible factors such that the sparseness of the unused factors is evident, as inferred via the posterior. The results in Figure 3 correspond to one example (representative) collection sample from the Gibbs sampler; Factor 1, which is most closely tied to the symptomatic/asymptomatic response, is employed by all data, while other factors are used more idiosyncratically (*e.g*., Factors 3 and 14 are only used by a small subset of the data samples; see the detailed discussion of the model in the Methods section).

At each time point, there are data from 17 subjects (the same individuals were sampled at a sequence of times). The horizontal axis in Figure 3 corresponds to a sequence of *groups *of data, proceeding in time from inoculation, with generally 17 samples per time point (all data will be released for other investigators to experiment with). The blue points correspond to samples of individuals who eventually became symptomatic, and the red points to asymptomatic individuals.

The vertical axis in these plots corresponds to the factor score associated with the respective sample. We observe in Figure 3 that Factor 1 (the factor indexing is arbitrary) provides a clear discriminator of those who will become symptomatic, particularly as time proceeds (note that the model is completely unsupervised, and therefore this discriminating power was uncovered without using label information).

**Figure 4:**
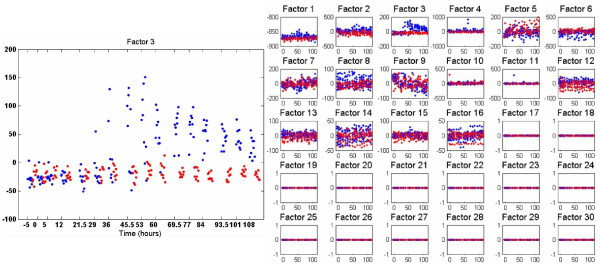
**Factor-analysis of Flu data with Bayesian pseudo-SVD applied within design of factor scores, applied to the Flu data**. Results are presented in the same form as Figure 3.

**Figure 5:**
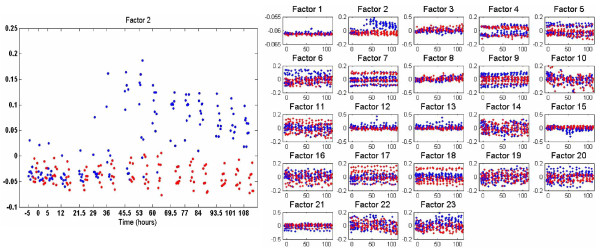
**Factor-analysis of Flu data with PMD **[13]**applied within design of factor scores, applied to the Flu data**. Blue points correspond to samples of individuals who ultimately become symptomatic, and the red points correspond to asymptomatic samples.

Having introduced the form of the data presentation, we now present results using the pseudo-SVD method and PMD; for the pseudo-SVD method we again show one (typical) sample from the Gibbs collection samples, while for PMD the results are the single solution. In Figures 4 and 5 we present results, respectively, for the Bayesian pseudo-SVD model and for PMD [13]. For the Bayesian methods we again set *K *= 30. Both methods uncover a relatively small (less than *K*) number of relevant factors.

Note that in each case there appears to be one factor that clearly distinguishes symptomatic vs. asymptomatic, particularly as time increases. Upon examining the important genes in each of these factors, one recognizes a high level of overlap (suggesting consistency between the different methods). Further discussion of the associated genes and their biological significance is provided in [25].

### E. Pan-viral factors

We now consider a "pan-viral" analysis, in which data from all three viruses are analyzed *jointly*. For further conciseness, for this example we only present results for the BP applied to the factor scores; similar results were obtained with the Bayesian pseudo-SVD framework and by PMD.

**Figure 6:**
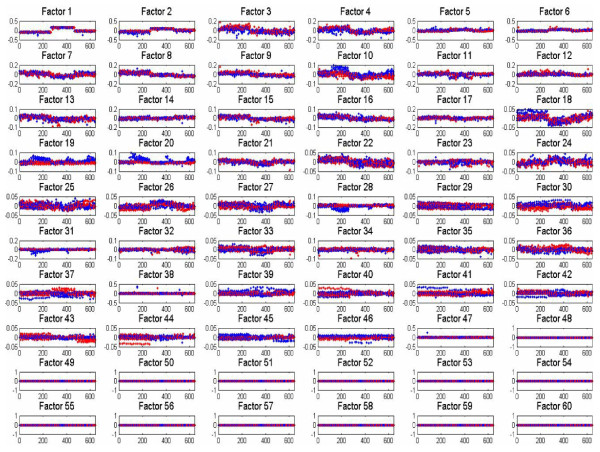
**Factor-analysis performed jointly to the Flu, RSV and Rhino data, with BP applied within design of factor scores, as discussed in Section IV-B**. Results are presented in the same form as Figure 3; the first 220 samples correspond to the Flu data, the next 210 samples correspond to Rhino virus, and the remaining samples correspond to RSV.

**Figure 7:**
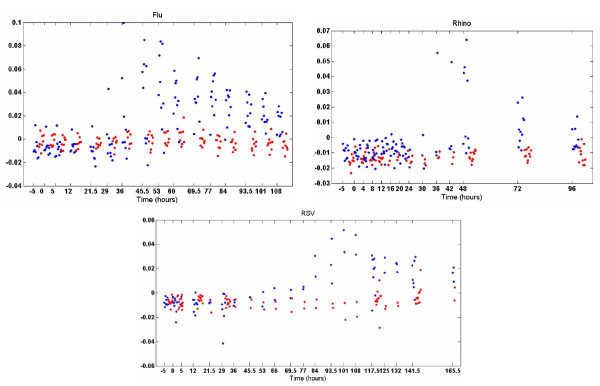
**Detailed view of factor 20 in Figure 6; blue points correspond to symptomatic subjects, and red to asymptomatic**. Influenza results are top-left, Rhino top-right, and RSV at bottom. The horizontal axis denotes time in hours. The data were collected in groups, at discrete times; the results at a given time are shifted slightly along the horizontal axis with respect to one another, to enhance readability.

Since three viruses are now considered jointly, we have increased *K *to *K *= 60 in this example, and now approximately 46 factors were inferred (with non-zero factor scores). Considering Figure 6, we note that Factor 20 provides good discrimination between the symptomatic (blue) and asymptomatic (red) samples, with this factor examined more closely in Figure 7. This same factor is able to distinguish the samples of each virus, at sufficient time after inoculation (a *single *"pan-viral" factor has been inferred, able to separately distinguish symptomatic vs. asymptomatic for each of the three viruses considered). Factor 19 in Figure 6 also appears to provide separation between symptomatic and asymptomatic samples; however, this is manifested because it contains two genes that are highly discriminative (SERPING1 and TNFAIP6), with most of the other genes in Factor 19 not discriminative. When addressing biological significance in [25], the focus is on Factor 20 in Figure 6, as it contains numerous discriminative genes. In these figures we are again showing one (typical) sample from the Gibbs collection.

It is also of interest to consider Factors 1 and 2 in Figure 6. Each of the samples from the individual viruses is offset by a distinct amplitude, almost entirely *independent *of whether the sample was symptomatic or asymptomatic. This phenomenon associated with Factors 1 and 2 in Figure 6 is attributed to challenge-study-dependent offsets in the data (the gene-expression values were obtained separately for each of these studies, and the data normalized separately), which account for different normalizations of the data between the three distinct viral challenges. This underscores that not all factors have biological significance, with some a consequence of the peculiarities of gene-expression data (study-dependent offsets in normalization). The other factor-analysis methods (omitted here for brevity) produced very similar normalization-related factors.

**Figure 8:**
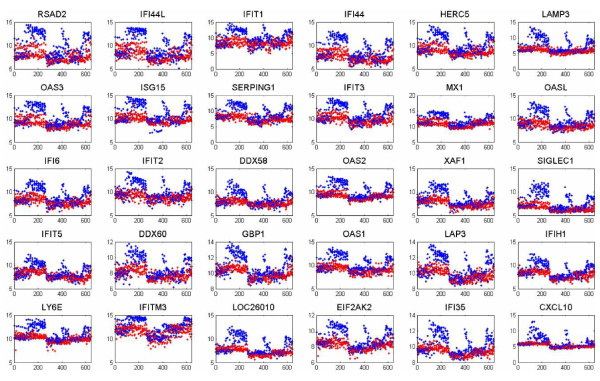
**Set of important genes inferred for combined analysis of Flu, RSV and Rhino data, associated Factor 20 from the BP applied to the factor scores (Figure 6)**. Blue points correspond to samples of individuals who ultimately become symptomatic, and the red points correspond to asymptomatic samples. The first 220 samples correspond to the Flu data (encompassing a total of 108 hrs), the next 210 samples correspond to Rhino virus (encompassing a total of 96 hrs), and the remaining samples correspond to RSV (encompassing a total of 165.5 hrs).

In Figure 8 are depicted the important genes associated with the discriminative pan-viral Factor 20 in Figure 6. It is a subject of further research, but based on the data analyzed thus far, it appears the FA model applied to gene-expression data cannot distinguish well *between *the different viruses. However, we have applied FA jointly to our pan-virus data and to bacterial data available from related but distinct studies [27]. From that analysis we are able to distinguish between viral-based phenotypes and bacteria-based phenotypes; this is discussed in greater detail in [25].

We have here identified many genes that are inferred to be connected with the viruses under study. It has been observed, by the medical doctors on our research team, that the inferred genes are closely aligned with relevant known pathways, with this discussed in detail in [25].

## III. Conclusions

We have examined two distinct but related objectives. First, in the context of Bayesian factor analysis, we have examined three ways of inferring an appropriate number of factors. Each of these methods is based on a different means of leveraging the utility of the Beta Process, and the closely related Indian Buffet Process (IBP). In the context of such models, we have examined inference based on variational Bayesian analysis, and based on a Gibbs sampler. We have also compared these Bayesian approaches to state-of-the-art non-Bayesian factor models.

The second contribution of this paper is the introduction of a new set of gene-expression data, from three time-evolving viral challenge studies. These data allow one to examine the time-evolution of Rhino virus, RSV and Influenza-A. In addition to the gene-expression data, we have also recorded clinical symptom scores, to which the gene-expression analysis may be compared. With the limited space available here, we have presented results on the Influenza data alone, and for all three viruses together (a "pan-viral" analysis).

Based on this study, we may make the following observations. For the number of Gibbs iterations deemed necessary, the VB and MCMC inference approaches required comparable computation time (VB was slightly faster, but not substantially). Although VB requires far fewer iterations (converges typically in 50 iterations), each VB iteration is significantly more expensive than that associated with MCMC. The advantage of using these two very distinct computational methods on the models considered is that they serve to cross-validate each other (providing confidence in the results, when these two very different methods agree, as they generally did in the studies considered).

Of the three methods of inferring the number of factors, the IBP applied to the factor loadings works well for small-scale problems, but it is computationally intractable for the large-scale viral data considered here. Applying the Beta Process to the factor scores, or to the singular values of a pseudo-SVD construction, yields reliable and high-quality results.

It is not our purpose to provide a detailed (perhaps philosophical) discourse on the relative merits of Bayesian and non-Bayesian approaches. However, we observed that the non-Bayesian Penalized Matrix Decomposition (PMD) yielded very high-quality results, as long as the model parameters were set carefully via cross-validation; very similar phenomenon was observed for the closely related sparse-PCA. Both PMD and sparse-PCA infer an appropriate number of factors, but one must very carefully set the stop criterion. Since PMD and sparse-PCA are much faster than the Bayesian approaches, perhaps a good compromise is to use the output of these models to initialize the Gibbs sampler in a Bayesian solution (this is a subject for future research).

Concerning the viral data, it was observed that all methods were able to infer a factor that was capable of distinguishing those subjects who would become symptomatic from those who would not. It was possible to infer a "pan-viral" factor, that was discriminative for all viruses considered.

The evolution of the factor scores tracked well the recorded clinical symptom scores. Further, for the discriminative factor, there was a good association between the genes inferred as important and the associated biology [25] (with interpretation provided by the medical doctors on our research team).

## IV. Methods

### A. Basic sparse factor model

Recall the factor model in (1); *r *defines the number of factors responsible for the data **X**, and it is not known in general, and must be inferred. Within the analysis we will consider *K *factors (*K *columns of A), with *K *set to a value anticipated to be large relative to *r*. We then infer the number of columns of **A **needed to represent the observed data **X**, with this number used as an estimate of *r*. Since we will be performing a Bayesian analysis, we will infer a posterior density function on *r*. Henceforth we assume **A **has *K *columns, with the understanding that we wish to infer the *r *<*K *columns that are actually needed to represent the data.

Let ***a**_k _*represent the *k*th column of **A**, for *k *= 1, . . . , *K*, and ***e**_j _*and ***s**_j _*represent respectively the *j*th columns of **E **and **S **(with *j *= 1, . . . , *n*). Within the imposed prior, vectors ***e**_j _*and ***s**_j _*are generated as *s_j _*~ N(0, ***I**_K_*), and $e_{j}\sim N(0,\text{diag}(\psi_{1}^{-1},\;\ldots ,\;\psi_{p}^{-1}))$ ; I*_K _*is the identity matrix and the precisions (*ψ*_1_, . . . , *ψ_p_*) are all drawn i.i.d. from a gamma prior.

One may consider many alternative means of defining sparseness on the ***a**_k_*, with the choice often dictated by convenience; we discuss two such methods here. In one approach [11] one may employ a spike-slab prior:

(2) $$A_{lk}\sim w_{lk}\delta_{0}+(1-w_{lk})N(0,\;\alpha_{k}^{-1})\text{ , }w_{lk}\sim \;Beta\;(a,\;b)\text{ , }\alpha_{k}\sim \;Gamma\;(c,\;d)$$

where (*a*, *b*) are selected as to strongly favor *w_lk _*→ 1, *δ*_0 _is a distribution concentrated at zero, and *l *= 1, . . . , *p*. The advantage of (2) is that sparseness is imposed explicitly (many components of ***a**_k _*are *exactly *zero).

An alternative to (2) is to employ a Student-t prior [6], implemented via the hierarchical construction

(3) $$A_{lk}\sim N(0,\;\alpha_{lk}^{-1})\text{ , }a_{lk}\sim \;Gamma\;(e,\;f)\;$$

but now with (*e*, *f*) selected as to constitute a Student-t sharply peaked about zero. One may employ a similar construction to impose a double-exponential (Laplace) sparseness-promoting prior [4].

### B. Beta process for inferring number of factors

The Beta Process (BP) was first developed by Hjort for survival data [28], and more recently it has found many other applications and extensions [19-21]. We here seek to provide a simple discussion of how this construction may be of interest in inferring an appropriate number of factors in factor modeling [22]. Our goal is to use the BP construction, which is closely related to the Indian buffet process (IBP) [19-21], to infer the number of factors *r *based on the observed data X.

Consider a measure drawn *H *~ BP(*α*, *β*, *H*_0_) and constructed as

(4) $$H=\sum_{k=1}^{K}\pi_{k}\delta_{a_{k}}\;\;\text{, }\pi_{k}\sim \text{Beta}(\alpha /\text{K},\;\beta (K-1)/K),\;\alpha_{k}\sim H_{0}$$

We seek to link our construction explicitly to the factor model, and therefore ***a**_k _*is the *k*th candidate factor loading (column of A), and *H*_0 _is defined by the construction in (2) or (3), depending upon which model is used. The expression *π_k _*represents the probability that ***a**_k _*is used to represent any particular data sample, defined by the columns of X. The expression *δ_ak _*represents a unit point measure concentrated at ***a**_k_*.

The BP is closely linked with a *Bernoulli Process *BeP(*H*) [21]. Specifically, for the *j*th column of **X**, we perform a draw from the Bernoulli process

(5) $$B_{j}=\sum_{k=1}^{K}z_{kj}\delta_{a_{k}},\;z_{kj}\in \{1,0\},\;z_{kj}\sim \text{Bernou}11\text{i}(\pi_{k})\;\;\;\;,\;\;\;j=1,\;\text{. }\text{. }\text{. , }n$$

where the *H *in BeP(*H*) is drawn *H *~ BP(*α*, *β*, *H*_0_), as defined in (4). As discussed further below, if *z_kj _*= 1 then ***a**_k _*is used as a factor loading to represent ***x**_j_*, the *j*th column of X; if *z_kj _*= 0, ***a**_k _*is not used to represent ***x**_j_*. In other words, *B_j _*is a sum of point measures (*δ_ak _*is a unit point measure concentrated at ***a**_k_*), and the binary variables *z_kj _*denote whether specific *δ_ak _*are employed within *B_j_*. More details on such constructions may be found in [21].

To make a connection to the introductory comments in Section II-A, and to relate the model to the IBP [19], we consider the above construction in the limit *K *→ ∞. Further, we marginalize (integrate) out the probabilities (*π*_1_, . . . , *π_K _*) used to constitute the BP draw *H*; we retain the *K *candidate factor loadings {***a**_k_*}_*k *= 1,*K *_used to define **A**, as drawn from the BP. Recall that ***x**_j _*represents the *j*th data sample (*j*th column of X). We assume that the data samples ("customers") select from among "dishes" at a "buffet", with the dishes defined by {***a**_k_*}_*k *= 1,*K *_. Data sample ***x***_1 _enters the buffet first, and selects the first *ν*_1 _dishes ***a***_1_, . . . , ***a**_ν_*_1 _, where *ν*_1 _is a random variable drawn from Possion(*α*/*β*). Therefore, the first column of **S **has the first *ν*_1 _elements as non-zero, with the remaining elements in that column set to zero. The second "customer" ***x***_2 _then enters the buffet, and selects from among the first *ν*_1 _dishes; the probability that ***x***_2 _selects ***a**_k_*, for each of *k *∈ {1, . . . , *ν*_1_}, is 1/(*β *+ 1); *i.e*., *z*_*k*2 _~ Bernoulli(1/(*β *+ 1)), for *k *∈ {1, . . . , *ν*_1_}. Customer ***x***_2 _also selects *ν*_2 _new dishes {***a**_ν_*_1+1 _. . . , ***a**_ν_*_1+*ν*2 _}, with *ν*_2 _**~ **Possion(*α */(*β *+ 1)). Hence, *z*_*k*2 _= 1 for *k *∈ {*ν*_1 _+ 1, . . . , *ν*_1 _+ *ν*_2_}, and unless stated explicitly otherwise, all other components of ***z**_j_*are zero. This process continues sequentially, with each ***x**_j _*entering the buffet in ascending order of *j*. Sample ***x**_J _*, with *J *∈ {1, . . . , *n*} selects dishes as follows. Let $C_{J-1}=\sum_{j=1}^{J-1}v_{j}$ represent the cumulative number of dishes selected off the buffet, among the previous customers {***x***_1_, . . . , ***x**_J_*_−1_}. Further, let *m*_*J*−1,*k *_≥ 1 represent the total number of times dish ***a**_k _*has been selected by previous customers {***x***_1_, . . . , ***x**_J_*_−1_}, for *k *∈ {1, . . . , *C*_*J*−1_}. Then ***x**_J _*selects dish ***a**_k_*, *k *∈ {1, . . . , *C*_*J*−1_}, with probability *m*_*J*−1,*k*_/(*β *+ *J *− 1); *i.e*., $z_{k,J}\sim \text{Bernoulli}\left(\frac{m_{J-1,k}}{\beta +J-1}\right)$ for *k *∈ {1, . . . , *C*_*J*−1_}. Note that the more "popular" ***a**_k _*among the previous *J *− 1 customers (*i.e*., larger *m*_*J*−1,*k*_), the more probable it is that it will be selected by ***x**_J _*. Additionally, ***x**_J _*selects new dishes ***a**_k _*for *k ***∈ **{*C*_*J*−1 _+ 1, . . . , *C*_*J*−1 _+ *ν_J_*}, where $v_{J}\sim \text{Poisson}\left(\frac{\alpha }{\beta +J-1}\right)$. Therefore we have *z_k, J _*= 1 for *k *∈ {*C*_*J*−1 _+1, . . . , *C*_*J*−1 _+ *ν_J_*}. Thus, each new customer selects from among the dishes (factor loadings) already selected by at least one previous customer, and the more "popular" one of these dishes is, the more probable it is that the new customer will select it. Further, a new customer will also select additional dishes (factor loadings) not selected by any of the previous customers. However, note that as *J *increases, the draws $v_{J}\sim \text{Poisson}\left(\frac{\alpha }{\beta +J-1}\right)$ are likely to be decreasing in size, since $\frac{\alpha }{\beta +J-1}$ is getting smaller with increasing *J*. Therefore, although *K *→ ∞, a *finite *subset of the candidate dishes (factor loadings) {***a**_k_*}_*k *= 1,*K *_will be used among the *n *customers, defined by the columns of **X**, thereby imposing sparseness in the use of factor loadings. This model is also fully exchangeable, in that the order of the columns of **X **may be permuted, with no change in the properties of the prior [19]. The model imposes that many of the *n *samples will share the same set of factors, but the model is flexible enough to allow idiosyncratic (sample-dependent) factor usage.

In practice *K *is finite, and therefore it is also if interest to consider the properties of this prior for finite *K*. For finite *K*, one may show that the number of non-zero components of ***z**_j _*is drawn from Binomial(*K*, *α */(*α *+ *β*(*K *− 1))), and therefore one may set *α *and *β *to impose prior belief on the number of factors that will be important. The expected number of non-zero components in ***z**_j _*is *αK*/[*α *+ *β*(*K *− 1)].

To complete the model specifications, note that ***a**_k _*from the Beta-Bernoulli construction above defines the *k*th column of the factor-loading matrix A. The factor-score matrix **S **utilizes the binary vectors ***z**_j _*= (*z*_1*j *_, . . . , *z_Kj_*)*^T ^*defined in (5), for *j *∈ {1, . . . , *n*}. Specifically, we define the *j*th column of **S **as $s_{j}={\hat{s}}_{j}\circ z_{j}$ (∘ represents a point-wise, or Hadamard product), with ${\hat{s}}_{j}\sim N(0,\;{\text{I}}_{K})$ . The vector product ${\hat{s}}_{j}\circ z_{j}$ selects a subset of the components in ${\hat{s}}_{j}$ , setting the rest to zero, and therefore the columns of **S **are sparse.

### C. Sparse factor modeling with BP/IBP placed on factor loadings

In the above discussion the Beta-Bernoulli and IBP processes were presented for a specific construction of the factor-analysis model, with the goal of making the connection to the model explicit and hence clearer. However, there are alternative ways of utilizing the IBP for design of factor models. Specifically, rather than using the binary vectors to construct **S**, as above, they may alternatively be used to define **A**, with factor scores designed as in traditional factor models. This approach was considered in [8], using an Indian Buffet Process (IBP) construction (explicitly using the marginalization discussed above). A limitation of this approach is that one must perform *p *draws from the IBP to construct **A**, and typically p is very large for the gene-expression problems of interest. When presenting results in Section II-B, we discuss our experience with this model on small-scale problems, although this approach was found computationally intractable for the motivating virus studies considered in Section II-D.

### D. Constructing pseudo singular values

The final Bayesian construction considered for inferring *r *is closely related to the non-Bayesian sparse-PCA [12] and penalized matrix decomposition (PMD) [13] models. We generate the vectors {***a**_k_*}_*k *= 1,*K *_as before, using a sparseness-promoting prior like that discussed in Section IV-A. Further, the factor scores ***ξ**_k _*for factor loading *k *is drawn ***ξ**_k _*~ N (0, **I***_n_*), for $k=1,\;\ldots ,\;K;\xi_{k}^{T}$ constitutes the *k*th row of **S**, and we consider *K *such rows, for large *K *(relative to the anticipated *r*). Finally, the vector of pseudo singular values **λ **= (λ_1_, . . . , λ*_K _*) is generated

(6) $$\begin{array}{l} \lambda \;=\;z\circ w\\ z_{k}\;\sim \text{ Bernou}11\text{i}(\pi_{k})\text{ , }k=1,\;\ldots ,\;K\\ \pi_{k}\;\sim \text{ Beta}(\alpha /\text{K},\;\beta K/(K-1))\text{ , }k=1,\;\ldots ,\;K\\ w\;\sim N(0,\;{\text{I}}_{K}) \end{array}$$

The matrix product AS in (1) is now constituted as $\sum_{k=1}^{K}\lambda_{k}a_{k}\xi_{k}^{T}$ . The non-zero components of **λ **select the columns of **A **used across all columns of X. As discussed in Section IV, the number of non-zero components of **λ **is drawn Binomial(*K*, *α*/(*α *+ *β(K *− 1))), and the posterior on the number of such components provides desired information on the number of factors *r*. Note that this construction is *like *the Beta-Bernoulli process discussed above, in that it utilizes *π_k _*~ Beta(*α*/*K*, *βK*/(*K *− 1)) and the Bernoulli distribution; however, it only draws the binary vector ***z **once*, and therefore there is not the idea of multiple "customers", as in the two IBP-related formulations discussed above.

### E. Computational issues, model quality and hyper-parameter settings

The MCMC results presented here correspond to using 5000 collection samples, after a burn-in of 2000 iterations. However, with 2000 burn-in iterations and 500 collection samples, the average results of the factor scores and factor loadings are almost identical to those found with 5000. For all MCMC results, we employed a singular value decomposition (SVD) of the data matrix to initialize the factor loading and factor score matrix in the FA model, as well as the right-and left-singular matrix in the matrix decomposition model. For each iteration of the Gibbs sampler a particular number of factors r are employed, and based upon all collection samples one may infer an approximate posterior distribution for *r*. Running on a typical modern PC, the computation times are summarized in Table 1 for the different models, as applied to the influenza data (using 100 VB iterations).

**Table 1:** Relative CPU times of the different models, implemented on a pc, as applied to the influenza data. the pmd method required a few minutes.

	CPU Time VB (hours)	CPU Time MCMC (hours)
BPFA	0.5	4.87
Bayesian Pseudo-SVD	0.11	3.47
FA in [11]	0.11	4.87

To be explicit, we provide detailed hyper-parameter settings for the model in (7)-(14); the other models are set similarly. Specifically, *α *= 1, *β *= 1, *c *= 1, and *d *= *g *= *h *= *e *= *f *= 10^−6^. These parameters were not optimized, and were set in the same manner for all experiments. Although the PMD model is a non-Bayesian method, it also has parameter settings that must be addressed carefully; two hyper-parameters need adjusting: the sparseness threshold and the stop condition [13]. In all PMD experiments, we set the sparseness threshold as 4, and the PMD iterations were terminated when the reconstruction error was smaller than 5%.

All calculations were performed on PCs with Intel Pentium Dual E2200 processors and 2.00 GB memory, and all software was written in Matlab. For the large-scale analysis performed on the real data discussed above, MCMC required approximately 4 hours of CPU, while VB required 3 hours (per analysis).

## Authors' contributions

The following authors performed the statistical analysis: BC, MC, JP, AH, JL, DD and LC. The following authors executed the three viral challenge studies, and performed all biological interpretation of the ressults: AZ, CW and GSG. All authors read and contributed to writing this paper.

## Appendix: Gibbs and Variational Bayesian Analysis

We here provide a concise summary of the inference methods applied to one of the Bayesian FA models discussed above, with this representative of the analysis applied to the rest. Specifically, we consider the model discussed in Section IV-B, in which the BP is applied within the factor-score matrix. The complete model may be expressed as

(7) $$x_{i}\;\sim N(A(z_{i}\circ s_{i}),\;\text{diag}(\psi_{1}^{-1},\;\ldots ,\;\psi_{p}^{-1}))$$

(8) $$z_{ki}\;\sim \;\text{Bernou}11\text{i}(\pi_{k})$$

(9) $$\pi_{k}\;\sim \text{ Beta}(\alpha /\text{K},\;\beta (K-1)/K)$$

(10) $$A_{jk}\;\sim N(0,\;\gamma_{jk}^{-1})$$

(11) $$s_{i}\;\sim N(0,\;\delta^{-1}{\text{I}}_{K})$$

(12) $$\gamma_{jk}\sim Gamma(c,\;d)$$

(13) $$\psi_{j}\sim Gamma(g,\;h)$$

(14) δ~Gamma(e, f)

where *i *= 1, . . . , *n*, *j *= 1, . . . , *p *and *k *= 1, . . . , *K*.

### Gibbs sampler

The full likelihood of the model is

$$\begin{array}{lll} p(X,\;Z,\;A,\;S,\;\psi ) & = & \prod_{i=1}^{n}N(x_{i};\;(A(z_{i}\circ s_{i})),\;\text{diag}{\left(\psi \right)}^{-1})N(s_{i};\;0,\;\delta^{-1}I_{K})\\ & \times & \prod_{j=1}^{p}\prod_{k=1}^{K}N(A_{jk};0,\;\gamma_{jk}^{-1})\text{Gamma}(\gamma_{jk};c,\;d)\\ & \times & \prod_{i=1}^{n}\prod_{k=1}^{K}\text{B}\text{ernou}11\text{i}(z_{ki};\;\pi_{k})\text{Beta(}\pi_{k}\;;\;\frac{\alpha }{K},\;\frac{\beta (K-1)}{K}\text{)}\\ & \times & \prod_{j=1}^{p}\text{G}\text{amma}(\psi_{j}\;;\;g,\;h)\times \text{Gamma}(\delta ;e,\;f) \end{array}$$

The sequential update equations of the Gibbs sampler are as follows.

• Sample each entry of the binary matrix, *z_ki_*. The probability of *z_ki _*= 1 is expressed as

$$p(z_{ki}=1|X,\;Z_{-ki},\;A,\;S,\;\psi )\propto \ln(\pi_{k})-\frac{1}{2}(A_{k}^{T}\text{diag}(\psi )A_{k}s_{ki}^{2}-2A_{k}^{T}\text{diag}(\psi )X_{i}^{-k}s_{ki}).$$

• Sample *π_k _*from *p(π_k_*|-) = Beta(*π_k_;α',β'*) where $\alpha \prime =\sum_{i=1}^{n}z_{ki}+\frac{\alpha }{K}$ and $\beta \prime =n+\frac{\beta (K-1)}{K}-\sum_{i=1}^{n}z_{ki}$.

• Sample each entry of factor loading matrix, *A_jk _*from *p(A_jk_*|−) = N (*A_jk_*; *μ_jk_*, Σ*_jk_*) where $\Sigma_{jk}={\left[\sum_{i=1}^{n}\psi_{j}s_{ki}^{2}z_{ki}^{2}+\gamma_{jk}\right]}^{-1},\;\mu_{jk}=\Sigma_{jk}(\sum_{i=1}^{n}\psi_{j}z_{ki}s_{ki}X_{ji}^{-k})$ , and $X_{ji}^{-k}=x_{ji}-\sum_{l=1,l\neq k}^{K}A_{jl}z_{li}s_{li}$.

• Sample each column of factor score matrix, ***s**_i_*, from *p(**s**_i_*|−) = N (*s_i_*; ξ*_i_*, Λ*_i_*) where $\lambda_{i}={\left[(A^{T}\circ {\tilde{Z}}_{i})diag(\psi )(A\circ {\tilde{Z}}_{i}^{T})+\delta {\text{I}}_{K}\right]}^{-1},\;\xi_{i}=\lambda_{i}(A\circ {\tilde{Z}}_{i})\text{diag}(\psi )x_{i}$, and ${\tilde{Z}}_{i}=[z_{i},\;\ldots ,\;z_{i}]$ with the *K*-dimensional vector,*z_i_*, repeated *p *times, 1 ≤ *i *≤ *n*.

• Sample *ψ_j _*from $p(\psi_{j}|-)=\text{Gamma}(\psi_{j}\;;\;g_{j}^{\prime },\;h_{j}^{\prime })$ where $g_{j}^{\prime }=g+\frac{n}{2}$ and $h_{j}^{\prime }=h+\frac{1}{2}\sum_{i=1}^{N}(\|x_{ji}-A_{j}(z_{i}\circ s_{i}){\|}^{2})$.

• Sample γ_jk _from *p*(γ*_jk_*|-) = Gamma (γ*_jk_*; *c', d'*) where *c' = c+*1/2 and $d\prime =d+\frac{1}{2}A_{jk}^{2}$.

• Sample *δ *from *p*(δ|-) = Gamma (δ*e', f'*) where *e' *= *e + nK/2 *and $f\prime =f+\frac{1}{2}\sum_{i=1}^{n}\left(s_{i}^{T}s_{i}\right)$ In the above equations expressions of the form *p(γ_jk_|*−) represent the probability of *γ_jk _*conditioned on all other parameters.

### Variational Bayesian inference

We seek a distribution Q(Θ; Γ ) to approximate the exact posterior p(Θ|**X**), where in Θ ≡{***A**,**S**,**Z**,α,π,ψ,γ,δ} *Our objective is to optimize the parameters Γ in the approximation Q(Θ; Γ). Toward that end, consider the variational expression

(15) $$\tilde{F}(\Gamma )=\int d\Theta Q(\Theta ;\Gamma )\ln\frac{Q(\Theta ;\Gamma )}{p(X)p(\Theta |X)}=-\ln p(X)+\text{KL}(Q(\Theta ;\Gamma )||p(\Theta |X))$$

Note that the term *p*(**X**) is a constant with respect to Γ, and therefore $\tilde{F}(\Gamma )$ is maximized when the Kullback-Leibler divergence *KL*(Q(Θ; Γ)||*p*(Θ|***X***)) is minimized. However, we cannot explicitly compute the KL divergence, since *p*(Θ|***X***) is unknown. However, the denominator term in $\tilde{F}(\Gamma )$ may be computed, since *p*(***X***)*p*(Θ|***X***) = *p*(***X***|Θ)*p*(Θ), and the prior *p*(Θ) and likelihood function *p*(***X***|Θ) are available. To make computation of $\tilde{F}(\Gamma )$ tractable, we assume *Q*(Θ|Γ) has a factorized form Q(Θ; Γ) = Π*_i_*Q*_i_*(Θ_i_; Γ*_i_*). With appropriate choice of *Q_i_*, the variational expression $\tilde{F}(\Gamma )$ may be evaluated analytically. The update equations are as follows.

• For *z_ki _*we have $Q(z_{ki})=\text{Bemou}11\text{i}(z_{ki};\;\rho_{ki}^{\prime })$ where $\rho_{ki}^{\prime }$ is the probability of *z_ki _*= 1. We consider the following two conditions:

discussion below, the symbol < • > represents the expectation of the argument.

$\ln Q(z_{ki}=1)\propto \zeta_{1}=\langle \ln\pi_{k}\rangle -[\frac{1}{2}(-2{\langle X_{i}^{-k}\rangle }^{T}\text{diag}(\langle \psi \rangle )\langle A_{k}\rangle \langle w_{ki}\rangle +\langle w_{ki}^{2}\rangle \langle A_{k}^{T}\text{diag}(\psi )A_{k}\rangle )]$$\ln Q(z_{ki}=0)\propto \zeta_{2}=\langle \ln(1-\pi_{k})\rangle$  where $X_{i}^{-k}=x_{i}-\sum_{l=1,l\neq k}^{K}A_{l}z_{li}s_{l}i,\;\langle \ln\pi_{k}\rangle =\Psi \left(\frac{\alpha }{K}+\sum_{i=1}^{n}\langle z_{ki}\rangle \right)-\Psi \left(\frac{\alpha +\beta (K-1)}{K}+n\right)$ ,$\langle \ln(1-\pi_{k})\rangle =\Psi \left(\frac{\beta (K-1)}{K}+n\;-\sum_{i=1}^{n}\langle z_{ki}\rangle \right)-\Psi \left(\frac{\alpha +\beta (K-1)}{K}+n\right),\;\Psi (x)=\frac{\partial }{\partial x}\ln\Gamma (x)$ and $\Gamma^{1}(x)=\int_{0}^{\infty }d\tau \tau^{x-1}e^{-\tau }$ . Therefore, we can calculate $\rho_{ki}^{\prime }=\frac{exp(\zeta_{1}))}{\exp(\zeta_{1})+exp(\zeta_{2}))}$ . Above, and in the discussion below, the symbol < • > represents the expectation of the argument.

• For *π_k _*we have $Q(\pi_{k})=\text{Beta}(\pi_{k}\;;\;\alpha_{k}^{\prime },\;\beta_{k}^{\prime })$ where $\alpha_{k}^{\prime }=\sum_{i=1}^{n}\langle z_{ki}\rangle +\frac{\alpha }{K}$ and $\beta_{k}^{\prime }=n+\frac{\beta (K-1)}{K}-\sum_{i=1}^{n}\langle z_{ki}\rangle$.

• For *A_jk _*we have $Q(A_{jk})=N(A_{jk};\mu_{jk},\;\Sigma_{jk})$ with $\Sigma_{jk}={\left[\sum_{i=1}^{n}\langle \psi_{j}\rangle \langle s_{ki}^{2}\rangle \langle z_{ki}\rangle +\langle \gamma_{jk}\rangle \right]}^{-1}$ and

$$\mu_{jk}=\Sigma_{jk}(\sum_{i=1}^{n}\langle \psi_{j}z_{ki}s_{ki}X_{ji}^{-k}\rangle$$

, where $X_{ji}^{-k}=x_{ji}-{\sum_{l=1;l\neq k}^{K}A}_{jl}z_{li}s_{li}$.

• For *s_i _*we have $Q(s_{i})=N(s_{i};\;\xi_{i},\;\lambda_{i})$ , with $\lambda_{i}={\left[\langle (A^{T}\circ {\tilde{Z}}_{i})\text{diag}(\langle \psi \rangle )(A\circ {\tilde{Z}}_{i}^{T})\rangle +\langle \delta \rangle I\right]}^{-1}$ and $\xi_{i}=\lambda_{i}((\langle A^{T}\rangle \circ \langle {\tilde{Z}}_{i}\rangle )\text{diag}(\langle \psi \rangle )x_{i})$ , where ${\tilde{Z}}_{i}=[z_{i},\;\ldots ,\;z_{i}]$ is a *K*-dimensional vector of all *zi *repeated *p *times. In order to exactly calculate the expectation,

$$B=\langle (A^{T}\circ {\tilde{Z}}_{i})\text{diag}(\psi )(A\circ {\tilde{Z}}_{i}^{T})\rangle$$

, we have to consider it as two parts. Specifically, the off-diagonal elements of ***B ***are $\left(\langle A^{T}\rangle \circ \langle {\tilde{Z}}_{i}\rangle )\text{diag}(\langle \psi \rangle )(\langle A\rangle \circ \langle {\tilde{Z}}_{i}^{T}\rangle \right)$ , and the diagonal elements, $B_{kk}=(\sum_{j=1}^{p}({\langle A_{jk}\rangle }^{2}+\Sigma_{jk})\langle \psi_{j}\rangle )\langle z_{ki}\rangle$ , since $\langle z_{ki}^{2}\rangle =\langle z_{ki}\rangle$ and $\langle A_{jk}^{2}\rangle ={\langle A_{jk}\rangle }^{2}+\Sigma_{jk}$ , where 1 ≤ *k *≤ *K*, 1 ≤ *j *≤ *p *and 1 ≤ *i *≤ *n*.

• For *ψ_j _*we have $Q(\psi_{j})=\text{Gamma}(\psi_{j}\;;\;g_{j}^{\prime },\;h_{j}^{\prime })$ , where $g_{j}^{\prime }=g+\frac{n}{2},h_{j}^{\prime }=h+\frac{1}{2}\sum_{i=1}^{n}\langle (x_{ji}-A_{j}{\left(z_{i}\circ s_{i}\right)}^{2}\rangle$.

• For *γ_jk _*we have $Q(\gamma_{jk})=\text{Gamma}(c_{jk}^{\prime },\;d_{jk}^{\prime })$ , with $c_{jk}^{\prime }=c+1/2\;$ and $d_{jk}^{\prime }=d+\frac{1}{2}\langle A_{jk}^{2}\rangle$.

• For *δ *we have *Q(δ*) Gamma (*e',f'*), where *e' = e + Kn/2 *and $f\prime =f+\frac{1}{2}\sum_{i=1}^{n}\langle s_{i}^{T}s_{i}\rangle$ .

## Acknowledgements

The research reported here was supported under the DARPA PHD program. The research and results presented here are the contributions of the authors, and the results were not influenced in any way by the sponsor.

## References

[B1] West M Bernardo JM, Bayarri M, Berger J, Dawid A, Heckerman D, Smith A, West M "Bayesian factor regression models in the "large p, small n" paradigm," Bayesian Statistics 7 2003 Oxford University Press 723 732

[B2] Tibshirani R "Regression shrinkage and selection via the lasso," Journal of Royal Statistical Society Ser. B 1996 58 267 288

[B3] Zou H Hastie T "Regularization and variable selection via the elastic net," Journal of Royal Statistical Society Ser. B 2005 67 301 320 10.1111/j.1467-9868.2005.00503.x

[B4] Park T Casella G "The Bayesian Lasso," Journal of the American Statistical Association 2008 103 681 686, 10.1198/016214508000000337

[B5] Cristianini N Shawe-Taylor J An Introduction to Support Vector Machines 2000 Cambridge University Press

[B6] Tipping M "Sparse Bayesian learning and the relevance vector machine," Journal of Machine Learning Research 2001 1 211 244 10.1162/15324430152748236

[B7] Ji S Xue Y Carin L "Bayesian compressive sensing," IEEE Transactions on Signal Processing 2008 56

[B8] Rai P Daum'e H III "The infinite hierarchical factor regression model," Proc Conf Neural Information Proc Systems (NIPS), Vancouver, Canada 2008

[B9] Knowles D Ghahramani Z "Infinite sparse factor analysis and infinite independent components analysis," 7th International Conference on Independent Component Analysis and Signal Separation 2007

[B10] Meeds E Ghahramani Z Neal R Roweis S "Modeling dyadic data with binary latent factors," Advances in Neural Information Processing Systems 2007 977 984

[B11] Carvalho C Chang J Lucas J Nevins JR Wang Q West M "High-dimensional sparse factor modelling: Applications in gene expression genomics," Journal of the American Statistical Association 2008 103 1438 1456 10.1198/016214508000000869 PMC3017385 21218139

[B12] Zou H Hastie T Tibshirani R "Sparse principal component analysis," Journal of Computational and Graphical Statistics 2006 15 2004 10.1198/106186006X113430

[B13] Witten D Tibshirani R Hastie T "A penalized matrix decomposition, with applications to sparse principal components and canonical correlation analysis," Biostatistics 2009 10 515 534 19377034 10.1093/biostatistics/kxp008 PMC2697346

[B14] Lopes H West M "Bayesian model assessment in factor analysis," Statistica Sinica 2004 14 41 67

[B15] Ghosh J Dunson D Dunson D "Bayesian model selection in factor analytic models," Random Effect and Latent Variable Model Selection 2008 John Wiley & Sons

[B16] Berger J Ghosh J Mukhopadhyay N "Approximation and consistency of Bayes factors as model dimension grows," J. Statist. Plann. Inference 2003 112 241258, 10.1016/S0378-3758(02)00336-1

[B17] Press S Shigemasu K "A note on choosing the number of factors," Comm Statist Theory Methods 1999 28 1653 1670 10.1080/03610929908832378

[B18] Lee S Song X "Bayesian selection on the number of factors in a factor analysis model," Behaviormetrika 2002 29 2339 10.2333/bhmk.29.23

[B19] Griffiths T Ghahramani Z "Infinite latent feature models and the indian buffet process," Advances in Neural Information Processing Systems 2005 475 482

[B20] Doshi-Velez F Miller K Gael JV The Y "Variational inference for the indian buffet process," AISTATS 2009

[B21] Thibaux R Jordan M "Hierarchical beta processes and the Indian buffet process," International Conference on Artificial Intelligence and Statistics 2007

[B22] Paisley J Carin L "Nonparametric factor analysis with beta process priors," Int Conf Machine Learning 2009

[B23] Beal M "Variational algorithms for approximate bayesian inference," 2003 Ph.D. dissertation, Gatsby Computational Neuroscience Unit, University College London

[B24] Zou H Hastie T Tibshirani R "Sparse principal component analysis," Technical Report, Statistics Department, Stanford University 2004

[B25] Zaas AK Chen M Lucas J Veldman T Hero AO Varkey J Turner R Oien C Kingsmore S Carin L Woods CW Ginsburg GS "Peripheral blood gene expression signatures characterize symptomatic respiratory viral infection," Cell Host & Microbe 2009 6 207 217 19664979 10.1016/j.chom.2009.07.006 PMC2852511

[B26] Dunson D "Dynamic latent trait models for multidimensional longitudinal data," J. Am. Statistical Ass 2003 98 555 563 10.1198/016214503000000387

[B27] Ramilo O Allman W Chung W Mejias A Ardura M Glaser C Wittkowski KM Piqueras B Banchereau J Palucka AK Chaussabel D "Gene expression patterns in blood leukocytes discriminate patients with acute infections," Blood 2007 109 2066 2077 17105821 10.1182/blood-2006-02-002477 PMC1801073

[B28] Hjort NL "Nonparametric bayes estimators based on beta processes in models for life history data," Annals of Statistics 1990 18 3 1259 1294 10.1214/aos/1176347749

